# The AKR1C3/AR‐V7 complex maintains CRPC tumour growth by repressing B4GALT1 expression

**DOI:** 10.1111/jcmm.15831

**Published:** 2020-09-09

**Authors:** Bin Wang, Shiqi Wu, Yong Fang, Guangxi Sun, Dalin He, Jer‐Tsong Hsieh, Xinyang Wang, Hao Zeng, Kaijie Wu

**Affiliations:** ^1^ Department of Urology First Affiliated Hospital of Xi'an Jiaotong University Xi'an China; ^2^ Department of Breast Surgery First Affiliated Hospital of Xi'an Jiaotong University Xi'an China; ^3^ Department of Urology The East Division of First Affiliated Hospital Sun Yat‐sen University Guangzhou China; ^4^ Department of Urology West China Hospital of Sichuan University Chengdu China; ^5^ Department of Urology University of Texas Southwestern Medical Center Dallas TX USA

**Keywords:** AKR1C3, AR‐V7, B4GALT1, castration‐resistant prostate cancer, complex

## Abstract

Multiple mechanisms contribute to the survival and growth of metastatic castration‐resistant prostate cancer (mCRPC) cells without androgen, including androgen receptor splice variants (AR‐V) and de novo intratumoral androgen synthesis. AKR1C3 is a critical androgenic enzyme that plays different roles in mCRPC, such as an EMT driver or AR coactivator. However, the relationship and regulatory mechanisms between AKR1C3 and AR‐V remain largely unknown. In this study, we observed a positive correlation between AKR1C3 and AR‐V7 staining in tissues from prostate rebiopsy at mCRPC. Mechanistically, AKR1C3 interacts with AR‐V7 protein in CRPC cells, which can reciprocally inhibit AR‐V7 and AKR1C3 protein degradation. Biologically, this complex is essential for in vitro and in vivo tumour growth of CRPC cells after androgen deprivation as it represses B4GALT1, a unique tumour suppressor gene in PCa. Together, this study reveals AKR1C3/AR‐V7 complex as a potential therapeutic target in mCRPC.

## 1 INTRODUCTION

Prostate cancer (PCa) is one of the most common cancers in the United States, which serves as the second leading cause of cancer‐related death in men.^1^ Most of cancer‐related deaths are associated with hormone‐resistant disease after androgen deprivation therapy (ADT). Recent studies have revealed several possible mechanisms related to the survival and growth of metastatic castration‐resistant PCa (mCRPC) cells without androgen, such as amplification of androgen receptor (AR), expression of constitutively active AR splice variants (AR‐V) and de novo intratumoral androgen biosynthesis.^2^, ^3^

The most widely studied AR‐V is AR‐V7. AR‐V7 is generated from aberrant alternative splicing of the third intron of AR pre‐mRNA. AR‐V7 is always expressed with full‐length AR (AR‐FL) in most cases, and its positive rate and intensity can be enhanced by hormonal therapies.^4^ Bryce et al reported that AR‐V7 can be detected in approximately 55% of patients after abiraterone treatment, and its expression can increase from 15% to 50% after enzalutamide treatment.^5^ Therefore, AR‐V7 has emerged as an important biomarker of resistance to these new‐generation anti‐androgen drugs in mCRPC. However, the mechanisms of its regulation and function have not been fully elucidated.

Aldo‐keto reductase 1C3 (AKR1C3), a pivotal androgenic enzyme, is highly expressed in metastatic and recurrent prostate xenografts and in sets of CRPC bone metastatic tumours acquired from patients in several studies.^6^, ^7^, ^8^ AKR1C3 has steroidogenic function, and patients with high AKR1C3 expression are often refractory to chemotherapy, radiotherapy and enzalutamide treatment.^9^ Our previous studies also revealed that AKR1C3 facilitates epithelial‐mesenchymal transition (EMT) and PCa metastasis, which indicates a poor prognosis of patients.^10^ Very recently, we have also demonstrated that AKR1C3 expression in primary lesion rebiopsy at the time of mCRPC is strongly associated with poor efficacy of abiraterone as a first‐line therapy.^11^ However, the mechanism of increased AKR1C3 leading to the failure of these novel hormonal therapies remains largely unknown.

In this study, we observed a positive correlation between AKR1C3 and AR‐V7 staining in tissues from prostate rebiopsy at mCRPC. Mechanistically, AKR1C3 bound to the AR‐V7 protein in CRPC cells, which reciprocally enhanced protein stability of AR‐V7 and AKR1C3 by inhibiting ubiquitin activity. Biologically, this complex was essential for in vitro and in vivo tumour growth of CRPC cells under ADT by uniquely repressing B4GALT1 expression. Consistently, B4GALT1 expression was significantly down‐regulated in PCa clinical specimens, especially in high‐grade, metastatic and recurrent PCa, which could be used to predict a poor prognosis of patients. Together, this study showed a new mechanism by which the AKR1C3/AR‐V7 complex regulates CRPC tumour growth by repressing a novel tumour‐suppressor gene.

## 2 MATERIALS AND METHODS

### 2.1 Cell culture

The human PCa cell lines (LNCaP, C4‐2, 22RV1 and VCaP) and the embryonic kidney cell line (HEK293) were kindly gifted by Dr Leland WK Chung (CedarsSinai Medical Center, Los Angeles, CA). Highly tumorigenic 22RV1 T sublines were generated from the isolated xenograft tumours of 22RV1 cells in athymic BALB/c nu/nu mice. 22RV1, 22RV1 T, VCaP and HEK293 cells were cultured in Dulbecco's modified Eagle's medium (DMEM, Invitrogen, Carlsbad, CA) with 10% fetal bovine serum (FBS). LNCaP and C4‐2 cells were cultured in T‐medium (Invitrogen) supplemented with 5% FBS. All these cells were maintained at 37℃ with 5% CO2.

### 2.2 Plasmids and cell transfection

The source and stable transfection of AKR1C3 shRNA and GFP‐AR‐V7 plasmids were described in our previous studies.^10^, ^12^ AKR1C3 shRNA plasmids were purchased from Taiwan RNA technology platform and gene manipulation core (http://rnai.genmed.sinica.edu.tw/index). Clone ID was TRCN0000278350 and TRCN0000026564. The vector was pLKO TRC005. Oligo sequence for AKR1C3 sh350 was CCGGCCGGAGTAAATTGCTAGATTTCTCGAGAAATCTAGCAATTTACTCCGGTTTTTG. Oligo sequence for AKR1C3 sh564 was CCGGCCGGAGTAAATTGCTAGATTTCTCGAGAAATCTAGCAATTTACTCCGGTTTTT. GFP‐AR‐V7 was kindly provided by Professor Jun Luo (Johns Hopkins University School of Medicine). The cDNA encoding the full length AR‐V7 (NM_001348061.1) was inserted into the pEGFP‐C3 vector to express the GFP‐AR‐V7 fusion protein. pcDNA3.1 AKR1C3 plasmid was kindly provided by Professor Allen Gao (UC Davis). To overexpress AKR1C3, LNCaP and 22RV1 cells were transfected with the empty vector pcDNA3.1 and pcDNA3.1 encoding AKR1C3, and both cell lines were maintained in medium with 300 mg/mL G418. Two B4GALT1 siRNAs (s5726, s5728; Thermo Fisher) and a control were purchased from Thermo Fisher Scientific (Shanghai, China). Transfections were performed using Lipofectamine 3000 (Invitrogen) according to the manufacturer's instructions.

### 2.3 Reagents and antibodies

MG132 and cycloheximide (CHX) were purchased from Selleck Chemicals (Houston, TX, USA). All antibodies used in this study are listed below: Mouse monoclonal Anti‐β‐actin (A1978, Sigma‐Aldrich, St. Louis, MO, USA), anti‐AKR1C3 (A6229, Sigma‐Aldrich), anti‐AR‐V7 (AG10008, Precision Antibody, Columbia, MD, USA), anti‐ubiquitin (sc‐47721, Santa Cruz, Santa Cruz, CA, USA), anti‐Bcl‐2 (sc‐7382, Santa Cruz); rabbit polyclonal anti‐AR (N‐20) (sc‐816, Santa Cruz) and anti‐B4GALT1 (A8546, ABclonal, Woburn, MA, USA).

### 2.4 Cell viability assay

The indicated cells were resuspended in 50 μl of DMEM with 10% FBS, plated in 96‐well plates at a concentration of 1500 cells/well and cultured for 24 hours and then recorded as 0 hours. Then, the medium was changed with phenol‐red free DMEM with 5% charcoal‐stripped FBS to mimic ADT conditions *in vitro*.^13^ After the indicated time of treatment, cell viability was analysed by a 3‐(4, 5‐dimethylthiazol‐2‐yl)‐2, 5‐diphenyltetrazolium bromide (MTT) assay (Roche, Indianapolis, IN, USA).

### 2.5 Colony formation assay

One thousand of the indicated cells were plated in 6‐well plates in duplicate and cultured in DMEM with 10% FBS in a humidified incubator containing 5% CO2 at 37°C. Cells were fed fresh phenol‐red free DMEM with 5% charcoal‐stripped FBS every 4 days for 2 weeks. Colonies were washed with phosphate‐buffered saline (PBS), fixed with 4% paraformaldehyde for 10 minutes and stained with 0.5% crystal violet for 5 minutes. Colonies were photographed, and visible colonies were counted.

### 2.6 RNA extraction and real‐time quantitative RT‐PCR

RNA was isolated using RNeasy Kit (FASTAGEN, CA, USA). cDNAs were reversely transcribed by the RevertAid kit (Takara, CA, USA), and a 25 µl system realtime PCRs were carried out in an iCycler thermal cycler (Bio‐Rad, Hercules, CA, USA) using SYBR Green Supermix (TAKARA, CA) with the gene‐specific primers: AKR1C3, F: 5’‐GAGAAGTAAAGCTTTGGAGGTCACA‐3’, R:5’ ‐CAACCTGCTCCTCATTATTGTATAAATGA‐3’; AR‐FL, F: 5’‐CTTACACGTGGACGACCAGA‐3’, R:5’‐GCTGTACATCCGGGACTTGT‐3; AR‐V7, F: 5’‐ CCATCTTGTCGTCTTCGGAAATGTTATGAAGC‐3’, R: 5’‐ TTTGAATGAGGCAAGTCAGCCTTTCT‐3’; SLC30A7, F: 5’‐ TCTCTTTCGCTTTTGTGGAACT −3’, R:5’‐ CCAGAACTTCCGCTCTAACATAC −3’; B4GALT1, F: 5’‐ CCAGGCGGGAGACACTATATT −3’, R:5’‐ CACCTGTACGCATTATGGTCAT −3’; HIF1A, F: 5’‐ GAACGTCGAAAAGAAAAGTCTCG −3’, R:5’‐ CCTTATCAAGATGCGAACTCACA −3’; SNX14, F: 5’‐ CCGCCTCCCTGCTTCTTAAC −3’, R:5’‐ CTGAAGTCCTAACTGCTTGGG −3’; 18S, F: 5’‐GGAATTGACGGAAGGGCACCACC‐3’, R:5’‐GTGCAGCCCCGGACATCTAAGG‐3’ (F, forward; R, reverse).

### 2.7 Western blot analyses

Cells were treated by RIPA buffer (150 mM NaCl, 50 mM Tris [pH 8.0], 1% NP40, 0.1% SDS, and 0.5% sodium deoxycholate) with 1% cocktail, proteinase inhibitors and 1 mmol/l PMSF (Sigma, St Louis, MO, USA). Then, 20 μg of protein was separated by 8%‐12% SDS‐PAGE and transferred to nitrocellulose membranes. Then, the membranes were blocked in Tris‐buffered saline with 0.1% Tween 20 and 5% skim milk for 1 hour. After blocking, the primary antibodies were used to incubate the membranes overnight at 4℃. After washing 3 times with TBST, membranes were incubated with horseradish peroxidase‐conjugated secondary antibodies for 1 hour at room temperature. Then, membranes were visualized by an ECL chemiluminescent detection system (Pierce, Rockford, IL, USA). A monoclonal β‐actin antibody was used to normalize the loading difference.

### 2.8 Immunoprecipitation assay

For immunoprecipitation, VCaP, 22RV1 T and HEK293 cells transfected with GFP‐AR‐V7 were washed twice with cold PBS and lysed in 1.5 ml of cold lysis buffer for 20 min on ice. The immunocomplex was first precipitated with Dynabeads Protein G (Life Technologies, Carlsbad, CA, USA) and then subjected to Western blot analysis as described previously.^13^

### 2.9 Immunofluorescence (IF)

22RV1 cells were transfected with GFP‐AR‐V7 and its control. Cells were washed with PBS, fixed in 4% paraformaldehyde, permeabilized in 0.5% Triton X‐100 and blocked in 5% bovine serum albumin (BSA). Cells were then incubated with primary AKR1C3 antibody at 4°C overnight and Alexa Fluor 633‐conjugated secondary antibodies, followed by nuclear staining with 5 μg/ml 4′,6‐diamidino‐2‐phenylindole (DAPI, Invitrogen). Signals were examined using a fluorescence microscope with glycerinum, and then a microscope was used to observe slides and take photos.

#### 2.9.1 Subcutaneous xenograft animal models

Male athymic BALB/c nu/nu mice were used according to protocols approved by the Ethical Committee of Xi'an Jiaotong University. All mice were castrated at the age of 4 weeks. One week later, mice were subcutaneously injected with 5 × 10^6^ 22RV1 sublines (shCon and sh564) in 100 μl medium containing Matrigel (1:1, v/v; BD Biosciences) into both flanks. The mice were killed 4 weeks after cell injection, and xenografts were stripped for measurement, Western blot analysis and immunohistochemistry (IHC) staining.

#### 2.9.2 Clinical specimens and IHC staining

All the prostate rebiopsies of mCRPC were obtained from the Department of Urology, West China Hospital of Sichuan University, China.^11^ All samples came from fine needle biopsy were used for IHC staining after written consent was obtained from patients. Due to the relatively small amount of tissue, each sample was enough to be taken one image. If the staining was positive in image, the whole sample was recorded as positive. IHC staining for clinical specimens and xenograft tumour tissues were carried out by the DAKO EnVision system. In brief, sections were deparaffinized, rehydrated and subjected to antigen retrieval in citrate buffer (10 mM, pH 6.0) for 5 min. Dual Block was used for 10 minutes to block endogenous peroxidase and alkaline phosphatase activities. The slides were then incubated overnight at 4℃ with the indicated primary antibodies. This step was followed by washing and incubation with EnVision secondary antibody for 30 minutes at room temperature. Signals were detected by adding hydrogen peroxide substrate using diaminobenzidine as a chromogen followed by haematoxylin counterstaining.

### 2.10 Bioinformatic data and statistical analysis

GEPIA (Gene Expression Profiling Interactive Analysis, http://gepia.cancer-pku.cn/) was used to analyse the RNA sequencing expression data of tumours and normal samples, the correlation of gene expression and patient survival analysis.^14^ All samples used for clinical statistical analysis were excavated from TCGA projects by using a standard processing pipeline. Statistical analyses were performed using GraphPad Prism version 5.0 (GraphPad Software, CA, USA) and SPSS 20.0 software (SPSS Inc, USA). Student's t test and Kaplan‐Meier analysis were used for comparisons as indicated. The Z‐score was calculated to normalize RNA expression. A *P* value <0.05 was considered statistically significant.

## 3 RESULTS

### 3.1 Positive correlation between AKR1C3 and AR‐V7 in CRPC tissues

**FIGURE 1:**
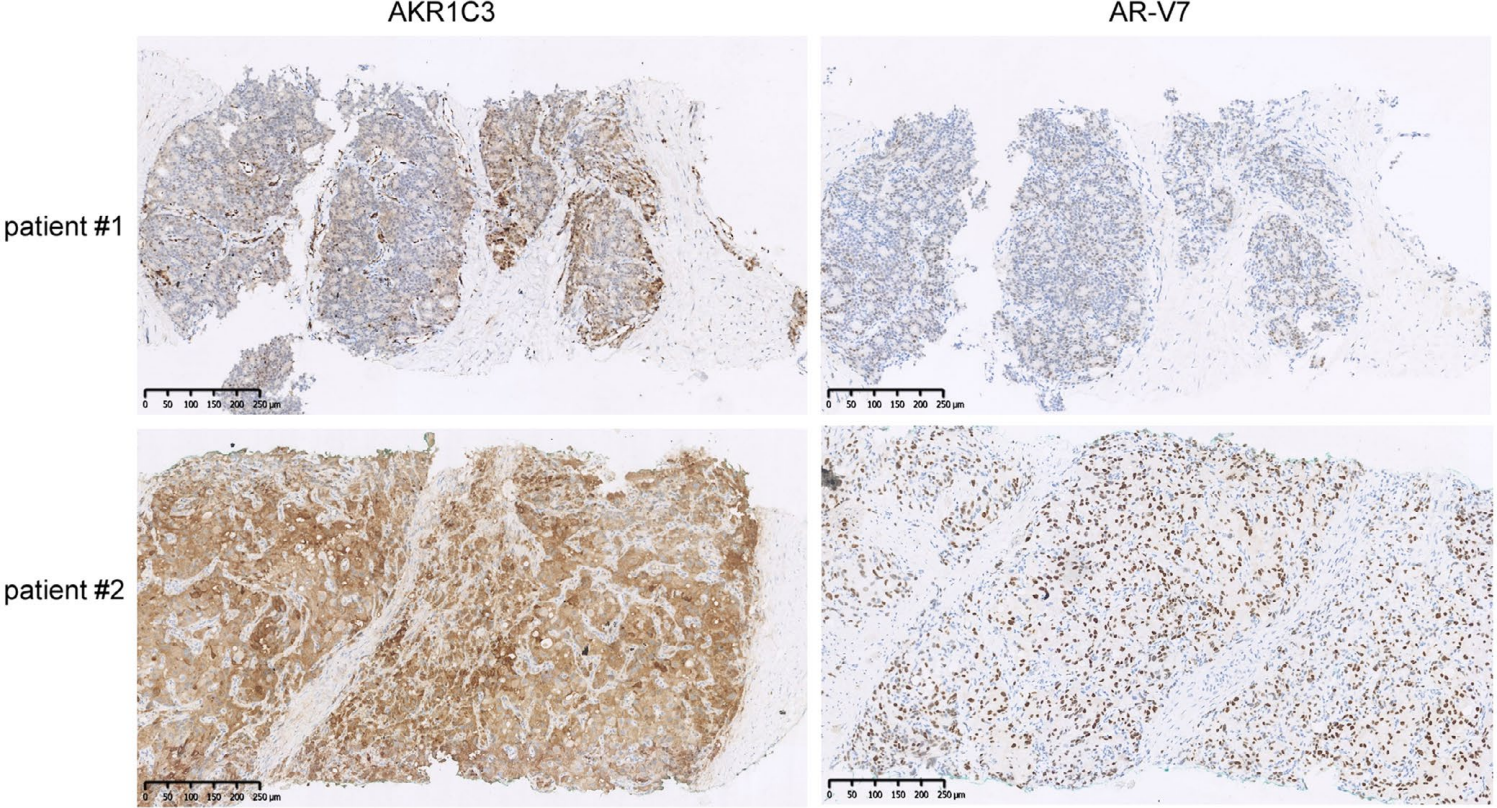
Expression of AKR1C3 and AR‐V7 in CRPC tissues. Representative pictures of AKR1C3 and AR‐V7 IHC staining in prostate re‐biopsy tissues at mCRPC. The scale bar is 250 μm. One image was taken per sample. 59/90 samples showed the representative pattern

As both AKR1C3 expression and AR‐V7 expression were increased in rebiopsy specimens at mCRPC compared with initial diagnosis in our previous study,^11^ we further investigated the correlation between AKR1C3 and AR‐V7 in CRPC tissues. As shown in Table 1, a significant reduction of AR‐V7 protein level was detected in AKR1C3‐negative patients compared with AKR1C3‐positive patients, while patient age, Gleason score, neuroendocrine differentiation, ECOG score, nadir T, ALP, LDH, HGB and baseline PSA showed no difference. Representative pictures of AKR1C3 and AR‐V7 staining were shown in Figure 1. Taken together, the positive correlation between AKR1C3 and AR‐V7 indicated their mutual regulation or interaction in the development of CRPC.

**TABLE 1:** The clinicopathological characteristics and IHC staining

	AKR1C3‐ (N = 53)	AKR1C3+ (N = 37)	*P* value
Age (Y)			
≥70, N (%)	29 (54.7%)	19 (51.4%)	0.753
<70, N (%)	24 (45.3%)	18 (48.6%)	
Gleason score			
ISUP 1, N (%)	5 (9.4%)	3 (8.1%)	0.898
ISUP 2, N (%)	11 (20.8%)	5 (13.5%)	
ISUP 3, N (%)	34 (64.2%)	27 (73.0%)	
ISUP 4, N (%)	1 (1.9%)	1 (2.7%)	
ISUP 5, N (%)	2 (3.8%)	1 (2.7%)	
Neuroendocrine differentiation			
Without, N (%)	46 (86.8%)	30 (81.1%)	0.462
With, N (%)	7 (13.2%)	7 (18.9%)	
ECOG			
0‐1, N (%)	45 (84.9%)	34 (91.9%)	0.319
>1, N (%)	8 (15.1%)	3 (8.1%)	
Nadir T (ng/mL)			
<0.1, N (%)	43 (81.1%)	33 (89.2%)	0.299
≥0.1, N (%)	10 (18.9%)	4 (10.8%)	
ALP (ng/mL)			
<160, N (%)	23 (43.4%)	18 (48.6%)	0.623
≥160, N (%)	30 (56.6%)	19 (51.4%)	
LDH (ng/mL)			
<215, N (%)	22 (41.5%)	20 (54.1%)	0.24
≥215, N (%)	31 (58.5%)	17 (45.9%)	
HGB (g/L)			
<120, N (%)	13 (24.5%)	8 (21.6%)	0.748
≥120, N (%)	40 (75.5%)	29 (78.4%)	
Baseline PSA (ng/mL)			
<50, N (%)	7 (13.2%)	7 (18.9%)	0.462
≥50, N (%)	46 (86.8%)	30 (81.1%)	
AR‐V7			
Negative, N (%)	46 (86.8%)	24 (64.9%)	0.014
Positive, N (%)	7 (13.2%)	13 (35.1%)	

Note

IHC, immunohistochemistry; ISUP, International Society of Urological Pathology; ECOG, Eastern Cooperative Oncology Group; ALP, Alkaline phosphatase; LDH, Lactate dehydrogenase; HGB, Haemoglobin; PSA, Prostate‐specific antigen; AR‐V7, Androgen receptor splice variant‐7.

### 3.2 AKR1C3 co‐localizes with and binds to the AR‐V7 protein

**FIGURE 2:**
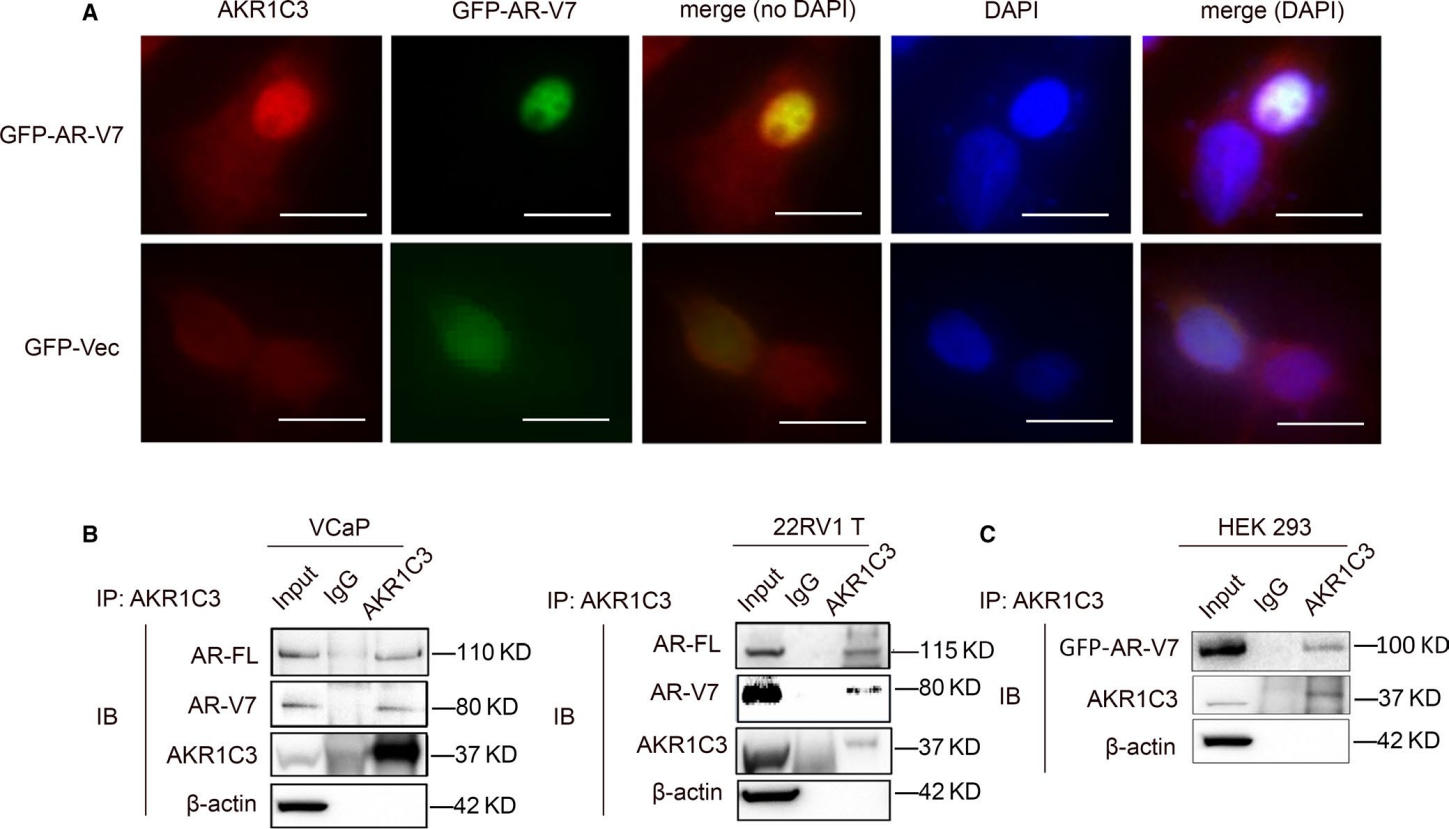
AKR1C3 co‐localizes and binds to AR‐V7 protein. A, after transfected with GFP‐AR‐V7 and vector control, 22RV1 cells were subjected to immunofluorescence of GFP‐AR‐V7 and staining with AKR1C3 antibody. The scale bar is 20 μm. Three images were taken per sample, and all the images show the representative pattern. Independent experiments were performed with similar results. B‐D, cell lysates of VCaP, 22RV1 and GFP‐AR‐V7 transfected HEK293 were immunoprecipitated with AKR1C3 antibody conjugated with protein G beads. Bound proteins were eluted and analysed by Western blot analysis. β‐actin was used as a loading control, and all the experiments were repeated at least 3 times

To determine the mechanism of regulation between AKR1C3 and AR‐V7, immunofluorescence was applied in 22RV1 cells transfected with GFP‐AR‐V7, and there was colocalization between AKR1C3 and AR‐V7 protein (Figure 2A). To further study this relevance, we used three cell lines to perform co‐immunoprecipitation. As both VCaP and 22RV1 express both endogenous AR‐V7 and AKR1C3, AR‐V7 was pulled down by using AKR1C3 antibody directly. HEK293 cells were transfected with exogenous AR‐V7 plasmid firstly because they only expressed endogenous AKR1C3. Indeed, the results showed that AKR1C3 could interact with both endogenous and exogenous AR‐V7 at the protein level (Figure 2B‐D).

### 3.3 AKR1C3 increases AR‐FL and AR‐V7 protein expression in CRPC cells

**FIGURE 3:**
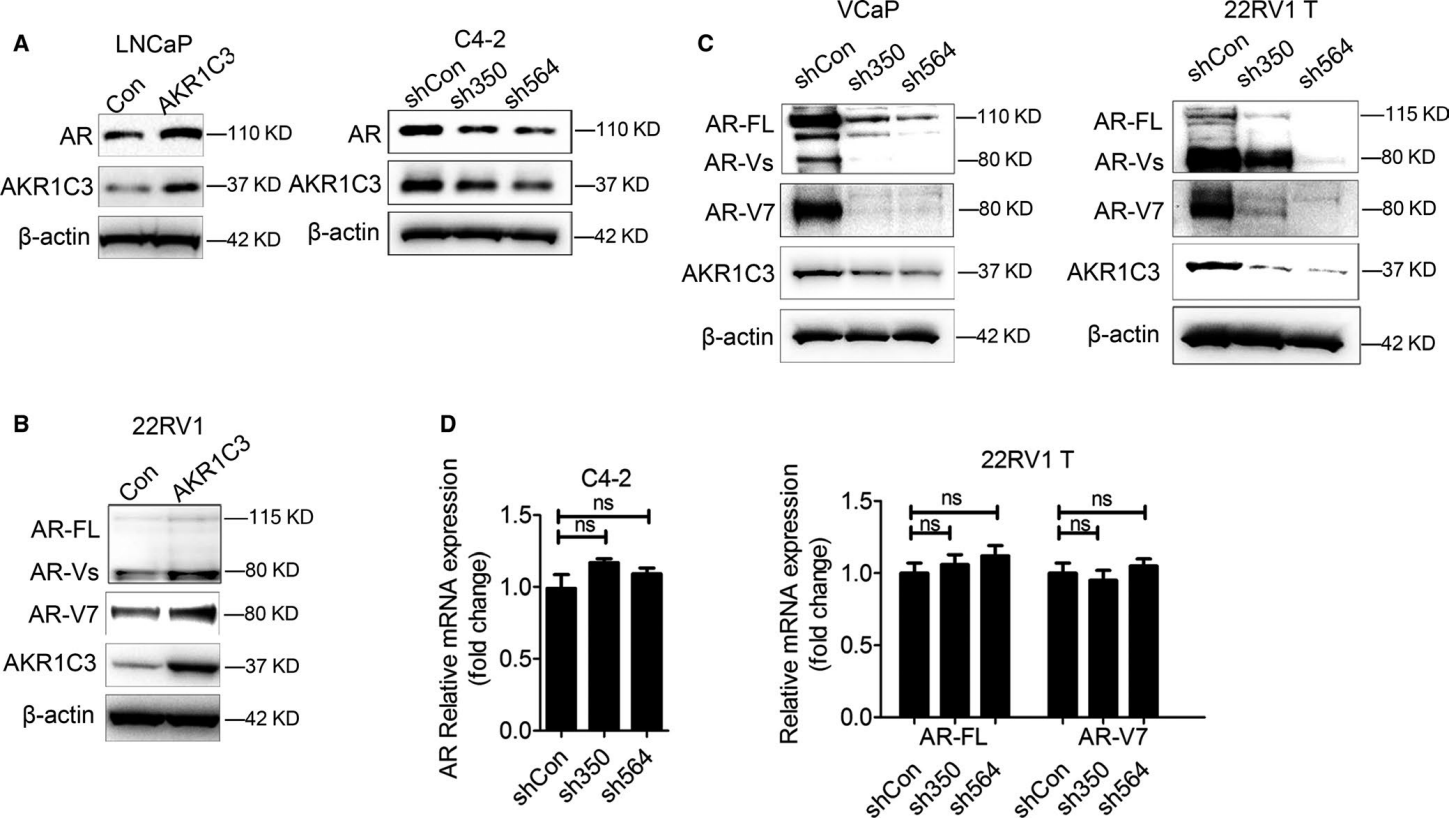
The effect of AKR1C3 on the expression of AR‐FL and AR‐V7 in PCa cells. A‐C, LNCaP, 22RV1 cells were transfected with AKR1C3 plasmids. C4‐2, VCaP and 22RV1 T cells were transfected with AKR1C3 shRNA lentivirus. AR‐FL or AR‐V7 protein expression was detected by Western blot analysis. β‐actin was used as a loading control, and all the experiments were repeated at least 3 times. D, C4‐2 and 22RV1 T cells were transfected with AKR1C3 shRNA lentivirus. The mRNA expression of AR‐FL and AR‐V7 was detected by RT‐PCR. 18s was used as a loading control, and all the experiments were repeated at least 3 times

To confirm the relationship between AKR1C3 and AR‐V7, AKR1C3 was overexpressed in LNCaP and 22RV1 cells, and the results showed that the expression of AR‐FL and AR‐V7 protein was up‐regulated (Figure 3A and B). Consistently, the expression of AR and AR‐V7 protein was down‐regulated by AKR1C3 knock‐down in C4‐2, VCaP and 22RV1 T cells (Figure 3A and C). However, the mRNA levels of AR‐FL and AR‐V7 showed no changes after AKR1C3 knock‐down in C4‐2 and 22RV1 T cells (Figure 3D). Together, these results showed that AKR1C3 increased AR‐FL and AR‐V7 expression at protein level.

### 3.4 AKR1C3 maintains both AR‐FL and AR‐V7 stability via the ubiquitin pathway

**FIGURE 4:**
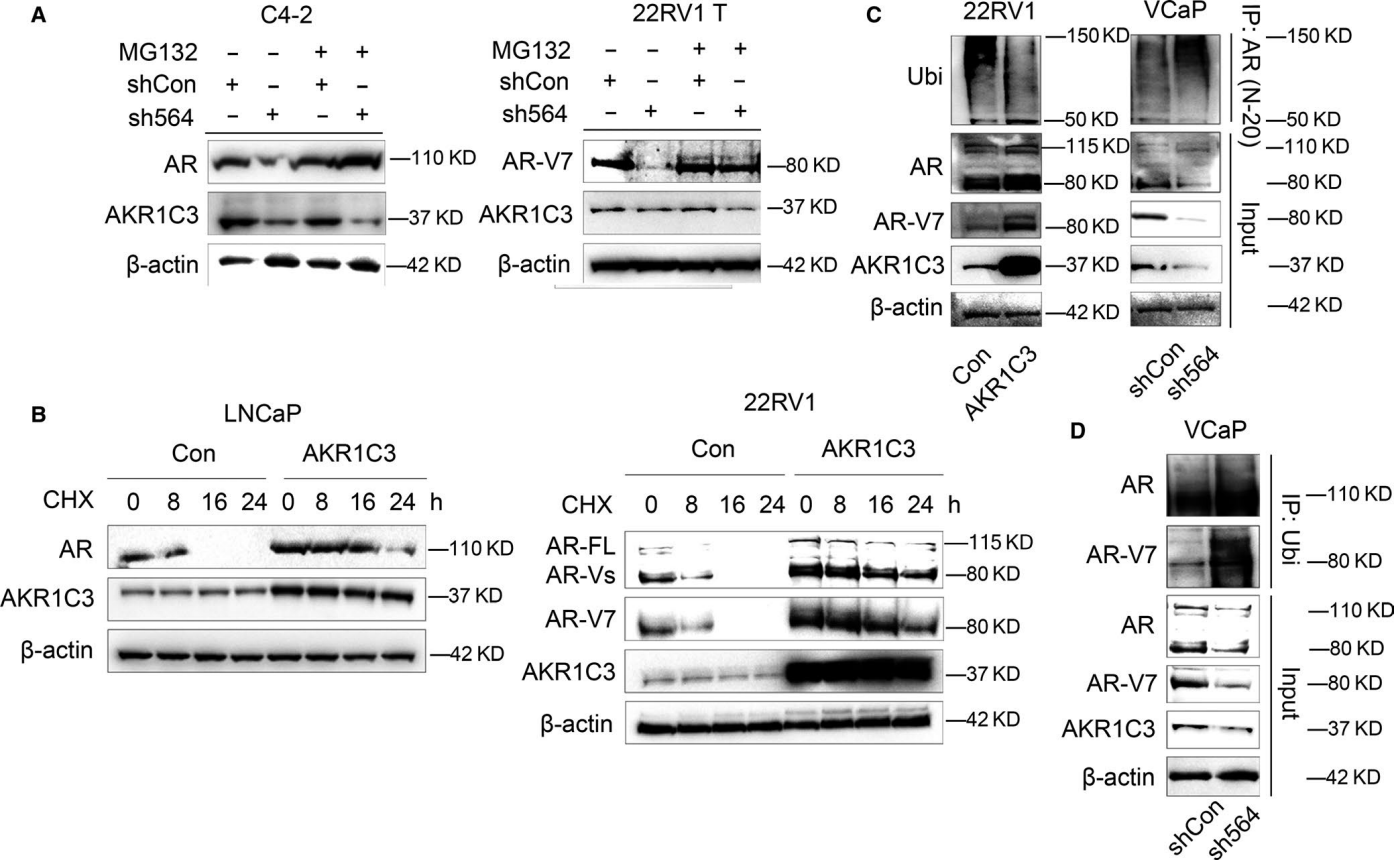
AKR1C3 maintains both AR‐FL and AR‐V7 stability by ubiquitin pathway. A, C4‐2 and 22RV1 T cells were treated with 10 μm MG132 for 8 h. AR‐FL, AR‐V7 and AKR1C3 proteins were detected by Western blot analysis. B, LNCaP and 22RV1 cells were treated with 15 μm CHX for 0, 8, 16, 24 hours. AR‐FL, AR‐V7 and AKR1C3 proteins were detected by Western blot analysis. C, after transfected with AKR1C3 plasmid or AKR1C3 shRNA lentivirus, cell lysates of 22RV1 and VCaP cells were immunoprecipitated with AR (N20) antibody conjugated with protein G beads. Bound proteins were eluted and analysed by Western blot analysis. D, VCaP cell lysates were immunoprecipitated with ubiquitin antibody conjugated with protein G beads. Bound proteins were eluted and analysed by Western blot analysis. β‐actin was used as a loading control, and all the experiments were repeated at least 3 times

To further elucidate mechanism by which AKR1C3 regulate protein expression of AR‐FL and AR‐V7. C4‐2 and 22RV1 T cells were treated with proteasome inhibitor MG132, and results showed that AKR1C3 maintained AR‐FL and AR‐V7 protein stability by down‐regulating the proteasome‐dependent degradation pathway (Figure 4A). LNCaP and 22RV1 cells with AKR1C3 overexpression were treated with the intracellular protein synthesis inhibitor CHX, and the results showed that the degradation of AR and AR‐V7 was significantly slowed down with overexpression of AKR1C3 (Figure 4B). As for the mechanism of AKR1C3 regulating AR‐V7 protein degradation, co‐immunoprecipitation was performed on 22RV1 and VCaP subline cells and the data showed that ubiquitin bound more AR‐FL and AR‐V7 for protein degradation when the expression level of AKR1C3 was low. (Figure 4C and D). Therefore, we also confirmed that AKR1C3 regulated AR‐FL and AR‐V7 protein degradation through ubiquitin pathway, which was consistent with a recent study reported by Liu et al^15^

### 3.5 AR‐V7 positively regulates the expression of AKR1C3

**FIGURE 5:**
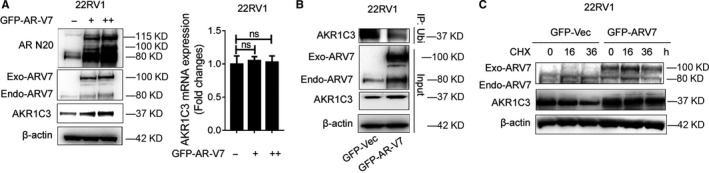
AR‐V7 enhances AKR1C3 protein expression. A, 22RV1 cells were transfected with GFP‐AR‐V7. AR, AR‐V7 and AKR1C3 proteins were detected by Western blot analysis. β‐actin was used as a loading control, and all the experiments were repeated at least 3 times. The mRNA expression of AKR1C3 was detected by RT‐PCR. 18s was used as a loading control, and all the experiments were repeated at least 3 times. B, after transfected with GFP‐AR‐V7 (exo‐ARV7) lentivirus, 22RV1 cell lysates were immunoprecipitated with ubiquitin antibody conjugated with protein G beads. Bound proteins were eluted and analysed by Western blot analysis. C, 22RV1 cells were transfected with GFP‐AR‐V7. 22RV1 GFP‐Vec and 22RV1 GFP‐AR‐V7 subline cells were treated with 15 μm CHX for 0, 16, 36 hours. AR‐V7 and AKR1C3 protein were detected by Western blot analysis

To better elucidate the regulation between AR‐V7 and AKR1C3, we further overexpressed exogenous AR‐V7 in 22RV1 cells. The result showed that transient overexpression of AR‐V7 up‐regulated AKR1C3 protein expression, but the mRNA level of AKR1C3 was not changed (Figure 5A). Co‐immunoprecipitation and CHX assays were also performed with 22RV1 sublines to show that AR‐V7 overexpression maintained AKR1C3 protein stability after CHX treatment by inhibiting ubiquitin‐mediated AKR1C3 protein degradation. (Figure 5B and 5C). The results demonstrated that the AKR1C3/AR‐V7 complex was also required to prevent AKR1C3 degradation at the protein level.

### 3.6 AKR1C3 maintains CRPC tumour growth after castration and regulates AR‐V7 expression in vivo

**FIGURE 6:**
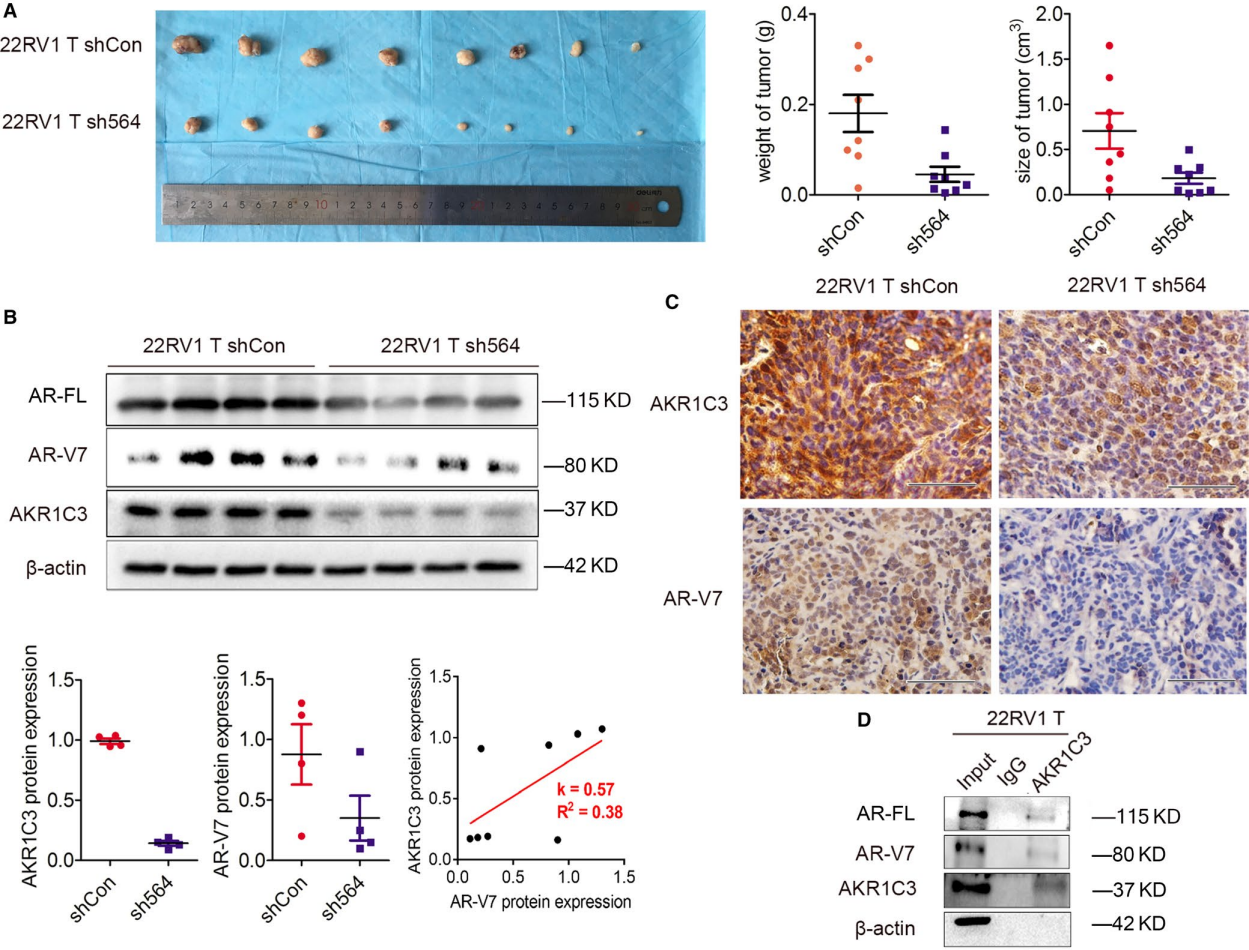
AKR1C3 maintains CRPC tumour growth after castration and regulates AR‐V7 expression in vivo. A, Pictures and quantification analysis of subcutaneous xenografts in nude mice injected with 22RV1 T/sh564 and 22RV1 T/shCon sublines (n = 8). Xenografts weight and size (cm^3^) were measured. B, AR‐FL, AR‐V7 and AKR1C3 proteins were detected in xenograft tissues from 4 mice by Western blot analysis. β‐actin was used as a loading control, and all the experiments were repeated at least 3 times. Linear regression was used to analyse the AKR1C3/AR‐V7 association in xenograft tumour tissue. C, Representative images of AKR1C3 and AR‐V7 IHC staining in subcutaneous xenografts. The scale bar is 50 μm. D, Cell lysates of subcutaneous xenografts injected with 22RV1 T were immunoprecipitated with AKR1C3 antibody conjugated with protein G beads. Bound proteins were eluted and analysed by Western blot analysis. β‐actin was used as a loading control, and all the experiments were repeated at least 3 times

To validate our in vitro data regarding the effects of AKR1C3 on tumour growth after castration and AR‐V7 expression, we generated subcutaneous xenograft model by injection of 22RV1 T sublines. Indeed, the tumour weight and size of 22RV1 T sh564 xenografts in castrated mice were significantly smaller than those of the controls (Figure 6A), indicating that AKR1C3 was required to drive CRPC tumour growth. Using these xenograft samples, we were able to validate the expression of AR‐V7 as well as AR‐FL. Western blot analysis and IHC staining in xenograft tumour tissue confirmed that the expression of AR‐V7 protein was decreased in 22RV1 T sh564 xenografts after knocking down AKR1C3, linear regression confirmed positive correlation between AKR1C3 and AR‐V7. AR‐FL expression was also diminished in AKR1C3‐silenced xenografts confirmed by Western blotting (Figure 6B and 6C). Consistent with in vitro data, co‐immunoprecipitation also confirmed that AKR1C3 could interact with both AR‐V7 and AR‐FL at the protein level in vivo (Figure 6D). The results showed that AKR1C3 maintained CRPC tumour growth after castration and regulated AR‐V7 expression in vivo.

### 3.7 The AR‐V7/AKR1C3 complex represses the transcription of B4GALT1

**FIGURE 7:**
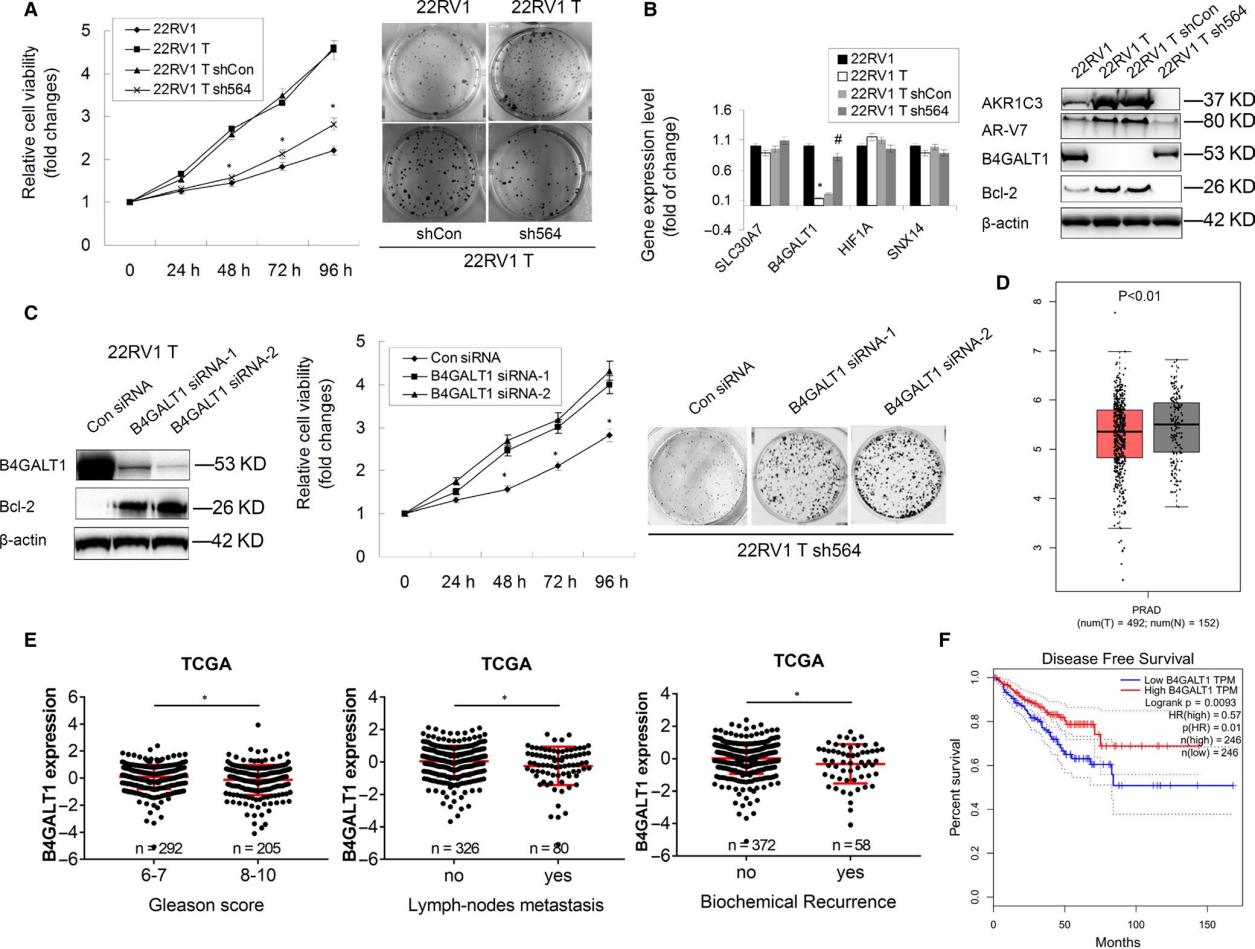
AR‐V7/AKR1C3 complex represses the transcription of B4GALT1 in CRPC. A, The cell proliferation and colonogenic ability of 22RV1, 22RV1 T and two 22RV1 T subline (shCon and sh564) cells were determined by colony formation assay and MTT assay, respectively. *P < .05. B, The mRNA expression of SLC30A7, B4GALT1, HIF1A and SNX14 was detected in 22RV1, 22RV1T, 22RV1 T shCon and 22RV1 T sh564 cells by RT‐PCR. 18s was used as a loading control. The expression of B4GALT1 and Bcl‐2 proteins was detected in these four cells by Western blot analysis. C, 22RV1 T sh564 cells were transfected with B4GALT1 specific siRNA and control. Protein expression of B4GALT1 and Bcl‐2 was determined by Western blot analysis, and cell proliferation and colonogenic ability were determined by MTT assay and colony formation assay, respectively. *P < .05. D, RNA‐sequencing data showing mRNA levels of B4GALT1 in tumour and normal tissues from TCGA. Unpaired t test was used to compare the differences of gene expression between normal (N = 152) and tumour (T = 492). E, RNA‐sequencing data showing mRNA levels of B4GALT1 classified by Gleason score, lymph‐node metastasis and biochemical recurrence in PCa tissues from TCGA. Unpaired t test was used to compare the differences of gene expression in each group. F, Kaplan‐Meier survival analysis of 492 patients with PCa from TCGA. 50% cut‐point was used to define low‐ or high‐expressed patients based on the mRNA levels of B4GALT1 gene. The long‐rank test was used to assess the differences between groups, and p value and hazard ration (HR) were shown

The functions of the AR‐V7/AKR1C3 complex on tumour growth were further studied by using MTT and colony formation assays in vitro. Indeed, 22RV1 T cells with high tumorigenic ability exhibited increased cell proliferation and colony formation abilities compared with parental 22RV1 cells, while knock‐down of AKR1C3 partially reversed this phenomenon (Figure 7A, *P* < 0.05). To further explore the potential downstream genes, we validated the expression of four AR‐V7 unique target genes (SLC30A7, B4GALT1, HIF1A and SNX14) that were previously reported.^16^ Interestingly, we only observed a significant decrease in B4GALT1 mRNA and protein expression in 22RV1 T cells compared with 22RV1 cells, while knockdown of AKR1C3 in 22RV1 T cells rescued this down‐regulation (Figure 7B). However, the RNA‐sequencing data from TCGA showed no significant correlation between AKR1C3 and B4GALT1 mRNA in tumour tissues (Figure S1). Furthermore, down‐regulation of B4GALT1 in 22RV1 T sh564 cells restored cell proliferation, colony formation and Bcl‐2 expression (Figure 7C, *P* < 0.05). Clinical data from the TCGA suggested that the expression of B4GALT1 was significantly lower in PCa tissues than in normal tissues (Figure 7D, *P* < 0.01). Consistently, PCa patients with higher Gleason scores, more metastatic lymph nodes and biochemical recurrence exhibited a lower expression of B4GALT1 (Figure 7E, *P* < 0.05), and these patients had worse disease‐free survival (Figure 7F, *P* = 0.0093). Collectively, these data suggested that the AR‐V7 target gene B4GALT1, as a novel tumour suppressor in PCa, was negatively regulated by AR‐V7/AKR1C3 complex.

## 4 DISCUSSION

Thirty percent of PCa patients have already developed metastatic lesions at diagnosis, and ADT has become one of the standard therapies for these patients. Unfortunately, PCa eventually recurs and develops into a lethal castration‐resistant disease. Multiple mechanisms lead to the survival and growth of CRPC cells without androgen, including AR mutation or amplification, altered expression of AR coactivators and corepressors, presence of AR‐V lacking the ligand‐binding domain (LBD) and de novo intratumoral androgen biosynthesis.^17^ In this study, we demonstrated that AKR1C3, a critical androgenic enzyme, formed a complex with AR‐V7 protein, and reciprocally inhibit AR‐V7 and AKR1C3 protein degradation, which led to concordant high AKR1C3 and AR‐V7 staining in CRPC tissues. Eventually, this complex enhanced the transcriptional repression of B4GALT1 gene expression by AR‐V7 serves as a novel growth‐suppressive gene in PCa.

AKR1C3 is an important enzyme in the steroidogenesis pathway that can produce androgen intratumorally to maintain CRPC tumour growth after ADT.^18^ Additionally, Liu et al demonstrated that it could contribute to drug resistance in patients treated with both abiraterone and enzalutamide, and inhibition of AKR1C3 activation with specific inhibitors (ie, indomethacin) could overcome these drug resistance by reducing the levels of intracrine androgens and diminishing AR transcriptional activity.^19^, ^20^ Very recently, we collected several primary prostate rebiopsies at the time of mCRPC diagnosis and performed IHC staining of AKR1C3. Indeed, AKR1C3 expression in CRPC tissues is strongly associated with poor efficacy of abiraterone as a first‐line therapy.^11^

In addition, AKR1C3 has been shown to play different roles in mCRPC, such as acting as an EMT driver or AR coactivator.^10^, ^21^ Our previous studies revealed that AKR1C3 could act as a novel EMT driver to facilitate PCa metastasis through ERK signalling, which indicated a poor prognosis of patients.^10^ Yepuru et al have reported that AKR1C3, as an AR‐selective coactivator, enhanced AR signalling through the ERG/AKR1C3/AR feed‐forward loop and facilitated CRPC development.^21^ Additionally, in our previous study, we generated several DAB2IP‐deficient PCa cells, which simultaneously exhibited activation of AR‐V7 and overexpression of AKR1C3, indicating a direct or indirect interaction between AKR1C3 and AR‐V7 protein. To further confirm their relationship in CRPC, we applied IHC staining of AR‐V7 in prostate rebiopsies at mCRPC. Indeed, there is a positive correlation between AKR1C3 and AR‐V7 staining. Mechanistically, AKR1C3 colocalized and bound to AR‐V7 protein in CRPC cells and then reciprocally inhibited AR‐V7 and AKR1C3 protein degradation, which led to concordant overexpression of AKR1C3 and AR‐V7. These results have also been partially confirmed by another group using different cell models. This group also reported that AKR1C3 promoted AR‐V7 protein stabilization and conferred resistance to AR‐targeted therapies.^15^

AR‐V7, as a truncated AR without a LBD, was believed to persist constitute AR target gene activation. However, Cato et al recently utilized cistrome and transcriptome analyses of CRPC data to show that AR‐V7 functions as a transcriptional repressor and heterodimerizes with full‐length AR at a subset of growth suppressive genes to support CRPC growth. Four genes, including SLC30A7, B4GALT1, HIF1A and SNX14, were specifically targeted by AR‐V7 and showed a negative effect on CRPC cell proliferation.^16^ Similarly, we demonstrated that only B4GALT1 transcription was suppressed by AR‐V7 in 22RV1 T cell models, and knock‐down of B4GALT1 restored the effect of the AKR1C3/AR‐V7 complex on 22RV1 T‐cell proliferation.

B4GALT1 is a member of the b‐1, 4‐galactosyltransferase gene (B4GALT) family and plays important roles in many biological events, including morphogenesis, mammalian fertilization, brain development and cellular adhesion. Recently, it was identified as a potential biomarker in malignancies with controversial effects. On the one hand, B4GALT1 serves as an oncogene in clear cell renal cell carcinoma, muscle‐invasive bladder cancer, lung adenocarcinoma, breast cancer and leukaemia.^22^, ^23^, ^24^, ^25^, ^26^, ^27^, ^28^ On the other hand, B4GALT1 is frequently methylated or down‐regulated in colorectal cancer and endometrial cancer^29^, ^30^ and can be used to predict patient outcomes. In this study, based on the analysis of TCGA database, we demonstrated that down‐regulated B4GALT1 in high‐grade, lymph‐node metastatic and biochemical recurrent PCa tissues predicts a poor prognosis of patients, indicating its role as a novel tumour suppressor in PCa.

**FIGURE 8:**
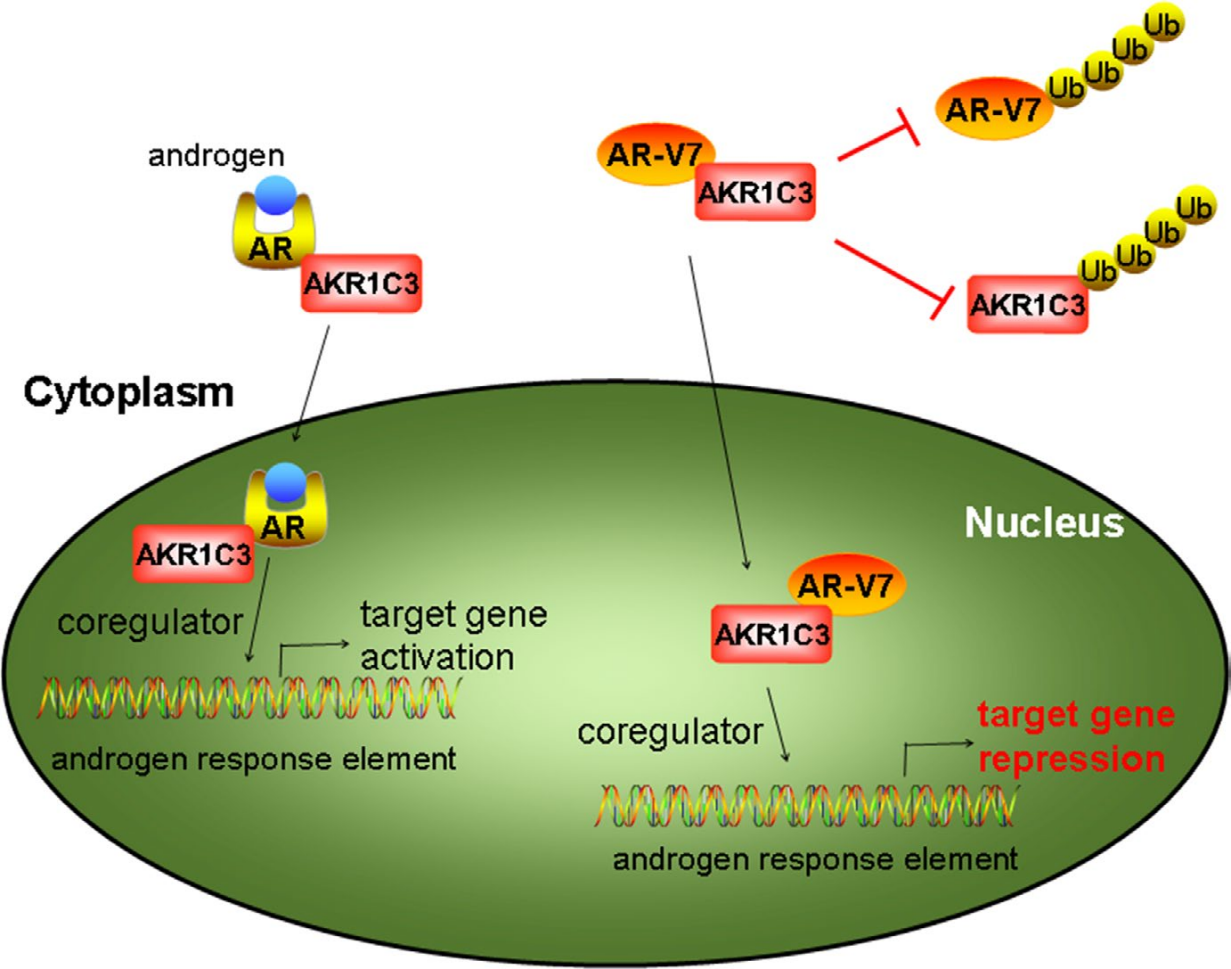
Schematic representation showing the role of AKR1C3/AR‐V7 complex by repressing B4GALT1 transcription in PCa

Taken together, this study identified AKR1C3 as a new critical regulator that increases the expression and transcriptional activity of AR‐V7 by forming a complex to inhibit its protein degradation. Additionally, this protein‐protein interaction can in turn to enhance AKR1C3 protein stability. This complex is essential for CRPC tumour growth as it represses the expression of the downstream B4GALT1 gene (schematic representation shown in Figure 8). Therefore, targeting AKR1C3/AR‐V7 complex might be an effective treatment strategy for patients with mCRPC.

## CONFLICT OF INTEREST

The authors declare no conflict of interest.

## AUTHORS’ CONTRIBUTION

Bin Wang: Conceptualization (equal); Data curation (equal); Formal analysis (equal); Methodology (equal); Resources (equal); Software (equal); Visualization (equal); Writing‐original draft (equal); Writing‐review & editing (equal). Shiqi Wu: Conceptualization (supporting); Data curation (equal); Formal analysis (supporting); Methodology (equal); Software (equal); Validation (equal); Writing‐original draft (equal). Yong Fang: Data curation (equal); Formal analysis (equal); Methodology (supporting); Validation (supporting); Visualization (equal). Guangxi Sun: Data curation (supporting); Formal analysis (equal); Software (equal); Validation (supporting). Dalin He: Conceptualization (supporting); Funding acquisition (supporting); Resources (equal); Supervision (equal). Jer‐Tsong Hsieh: Conceptualization (equal); Resources (equal); Supervision (equal). Xinyang Wang: Methodology (supporting); Resources (equal). Hao Zeng: Software (equal). Kaijie Wu: Conceptualization (equal); Funding acquisition (lead); Project administration (equal); Supervision (lead); Validation (equal); Visualization (equal); Writing‐original draft (equal); Writing‐review & editing (equal).

## Supporting information

Fig S1

Supplementary Material
