# Corrigendum to “The AKR1C3/AR‐V7 Complex Maintains CRPC Tumour Growth by Repressing B4GALT1 Expression”

**DOI:** 10.1111/jcmm.70885

**Published:** 2025-10-17

B. Wang, S. Wu, Y. Fang, G. Sun, D. He, J. T. Hsieh, X. Wang, H. Zeng, and K. Wu. “The AKR1C3/AR‐V7 Complex Maintains CRPC Tumour Growth by Repressing B4GALT1 Expression.” *Journal of Cellular and Molecular Medicine* 24, no. 20 (2020): 12032–12043. https://doi.org/10.1111/jcmm.15831.

In the article, there were errors in Figure 2C and Figure 3B. The correct Figure 2C and Figure 3B are shown below.

Figure 2C

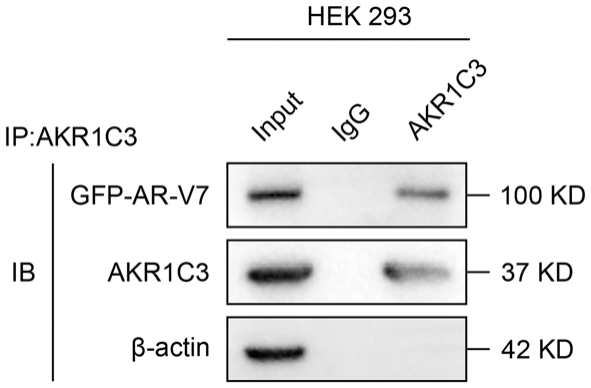

Figure 3B

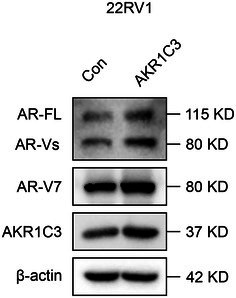

We apologise for this error.

