# Supplementary figures and images for: The AKR1C3/AR‐V7 complex maintains CRPC tumour growth by repressing B4GALT1 expression
Source: J Cell Mol Med. 2020 Sep 9;24(20):12032–43. doi: 10.1111/jcmm.15831 (PMC7579719; doi:10.1111/jcmm.15831)

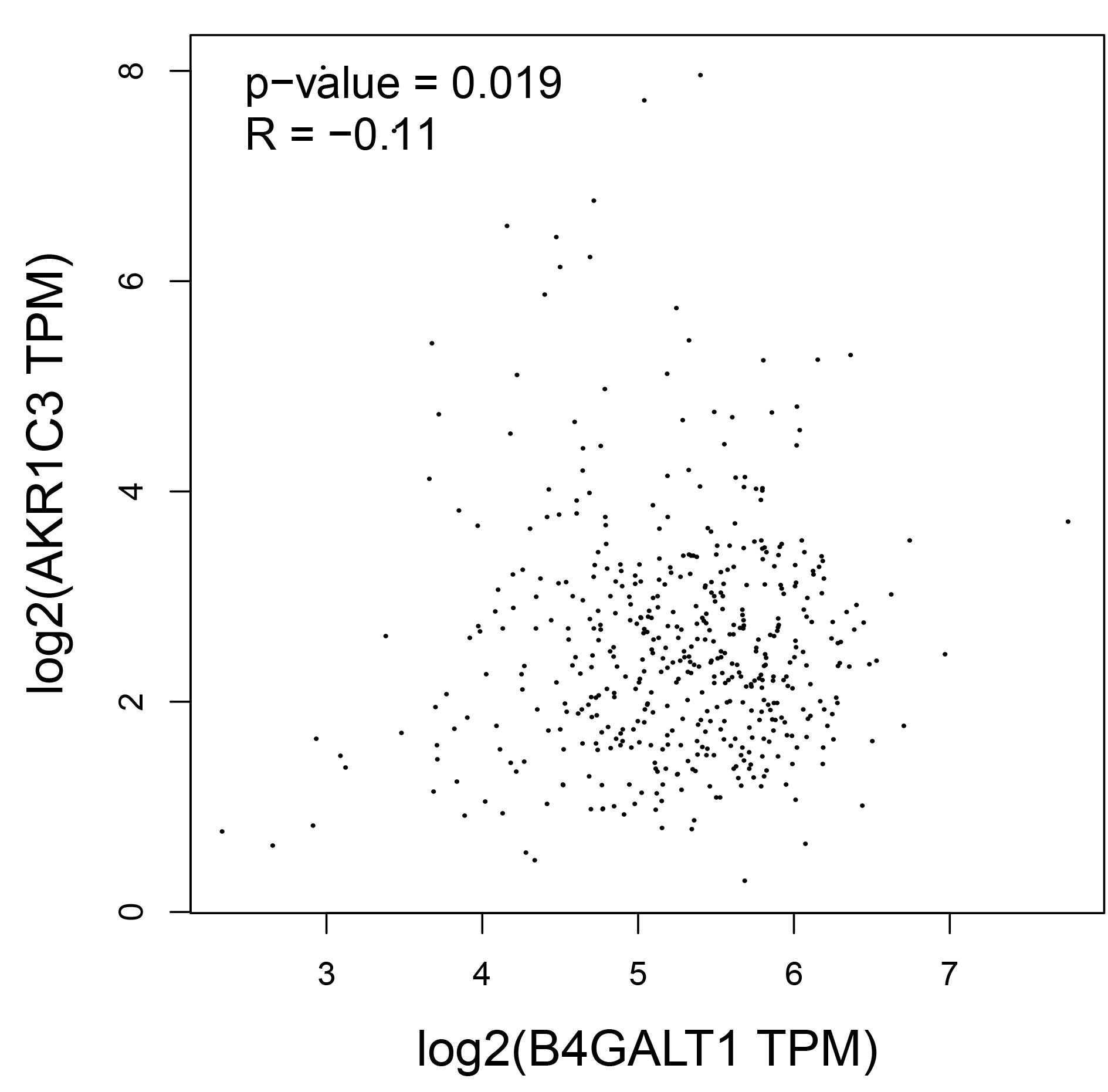

Supplement: Supplementary file 1 — Fig S1 [file JCMM-24-12032-s001.tif]
