# Supplementary material for: The AKR1C3/AR‐V7 complex maintains CRPC tumour growth by repressing B4GALT1 expression
Source: J Cell Mol Med. 2020 Sep 9;24(20):12032–43. doi: 10.1111/jcmm.15831 (PMC7579719; doi:10.1111/jcmm.15831)
Supplement: Supplementary file 2 — Supplementary Material [file JCMM-24-12032-s002.docx]

**Supplementary Figure 1. The correlation between AKR1C3 and B4GALT1 mRNA expression in human prostate cancer database.**

Normalized RNA-sequencing data showing the mRNA levels of AKR1C3 and B4GALT1 in tumor tissues from TCGA. Linear regression was used to analyze the correlation between AKR1C3 and B4GALT1 mRNA expression.
